# Effect of instrument-assisted soft tissue mobilization combined with blood flow restriction training on function, pain and strength of patients with patellofemoral joint pain

**DOI:** 10.1186/s12891-023-06701-6

**Published:** 2023-08-31

**Authors:** Yang Liu, Lianqing Wu

**Affiliations:** Wuhan Institute of Sports, No. 461 Luoyu Road, Hongshan District, Wuhan, Hubei Province China

**Keywords:** Instrument-assisted soft tissue mobilization, Patellofemoral pain syndrome, Blood flow restriction training

## Abstract

**Background:**

Patellofemoral pain syndrome is a prevalent sports injury that affects athletes both in their daily lives and during training. This condition causes pain in the area where the kneecap and thigh bone meet, and it can be quite debilitating. Whether an athlete is simply going about their day or pushing themselves to the limit during a workout, patellofemoral pain can be a significant hindrance.

**Purpose:**

The purpose of this study is to investigate the impact of combining Instrument-Assisted Soft Tissue Mobilization (IASTM) treatment with blood flow restriction training on individuals with patellofemoral pain. Specifically, the study will assess improvements in pain levels, functional ability, strength, and joint mobility resulting from this treatment approach.

**Methods:**

Twenty-six patients diagnosed with patellofemoral pain were selected as observation subjects and randomly divided into two groups: the IASTM combined with blood flow restriction training treatment group (*n* = 13) and the IASTM treatment group alone (*n* = 13). The treatment period was 4 weeks. In this study, we conducted a comparison and analysis of the knee’s visual analogue pain scale (VAS), Lysholm score, and a modified version of the Thomas test (MTT) at three different time points.In this subject paper, we compared and analyzed the VAS score of the knee, Lysholm score of the knee, and MTT at three different time points—before treatment, immediately after the first treatment, and after four weeks of treatment. Additionally, we recorded data using a maximum isometric muscle strength testing system for the lower extremity extensors four weeks before and after treatment.

**Results:**

In comparing the Lysholm scores within the groups, a significant difference was observed between the two groups following the initial treatment and after 4 weeks of treatment (*p* < 0.05). The scores increased, indicating a significant improvement in function. The VAS scores significantly differed after the first treatment and 4 weeks of treatment compared to before treatment (*p* < 0.05), indicating a significant improvement in pain. Additionally, after 4 weeks of treatment, the strength of the extensor muscle in the lower extremity significantly improved (*p* < 0.001). However, there was no significant difference in the strength test between the groups (*p* > 0.05). The MTT test revealed significant changes in the three joint angles before and after treatment (*p* > 0.05), suggesting an improvement in joint mobility. Overall, these results demonstrate the effectiveness of the treatment in improving pain and muscle strength in the lower extremity.

**Conclusion:**

The combination of IASTM treatment and blood flow restriction has been shown to significantly reduce pain and improve periprosthetic soft tissue flexibility. Additionally, IASTM treatment alone was found to be more effective in improving knee pain and muscle flexibility, ultimately leading to increased knee strength in a pain-free state. In terms of the overall treatment outcome, it was found that the combined treatment was significantly more effective than the adjuvant soft tissue release treatment alone.

## Introduction

Patellofemoral pain syndrome (PFPS) is a prevalent cause of knee pain in clinical settings. This condition is typically identified by anterior knee pain during physical activities, including walking, running, jumping, and stair climbing [1]. The development of PFPS is linked to an abnormal patellofemoral trajectory and increased local stress on the joint [2]. Various factors contribute significantly to the development of this condition, with abnormal patellar motion trajectory being a primary cause. This condition is often linked to lower limb force line disorders, which can result from a range of factors such as abnormal lower limb structural anatomy, weakened lower limb muscles, muscle tone imbalances around the knee joint, and sports-related injuries. In the meantime, Sinaei et al. [3] have demonstrated that the weakened strength of the biceps femoris muscle, delayed activation of the medial femoral muscle, and over-activation of the lateral femoral muscle relative to the medial test may be the root cause of PFPS symptoms. The above information highlights the significance of muscle tone imbalance in the muscle tissue surrounding the knee joint as a leading cause of pain. Therefore, this study aims to address the issue of dystonia imbalance in patients with patellofemoral pain syndrome (PFPS) through conservative treatment [1, 4]. Specifically, the study will utilize Instrument-Assisted Soft Tissue Mobilization (IASTM) therapy and blood flow restriction compression training to improve the symptoms of these patients.

IASTM treatment is a non-invasive therapeutic technique that uses specialized tools to externally intervene and mobilize soft tissues on the body surface. In this experiment, the fascial knife was utilized as a treatment tool to alleviate symptoms of pain and abnormal tension in the myofascia [5]. There are five main knife types, including Type M—Big M, Type A—Shark, Type S—Hook, Type C—Probe, and Type B—Bat, and depending on the particular shape of each knife they have different functional uses.

This treatment allows for quick identification and removal of fascial adhesions, effectively preventing soft tissue fibrosis and muscle degeneration [6, 7]. Fascial knife technology, also referred to as the fascial spatial balancing technique, is a treatment method for joint problems [8, 9]. This technique involves a combination of assessment, screening, and a special knife technique that applies pressure to the skin. The entire process is painless and safe. By targeting high-stress fascia, this technique can effectively improve joint range of motion [9]. This study aimed to improve knee mobility by releasing the overstressed muscles and tendons around the knee joint through IASTM treatment.

Blood Flow Restriction (BFR) training has been a widely-used technique in musculoskeletal pain rehabilitation for some time. It has proven particularly effective in clinical rehabilitation of lower extremity knee disorders, where it can significantly improve muscle atrophy, strengthen muscle strength and lower extremity endurance, and reduce pain and associated symptoms. As a result, BFR training can greatly enhance patients’ motor ability. BFR training can be used for various target groups when combined with lower load resistance or aerobic training [10–12]. The training mechanism is to control the blood circulation of the limbs by applying a certain mechanical pressure to the proximal end of the limb with a bandage or cuff while the patient is at rest or exercising, thus improving the training efficiency, muscle strength and nerve recruitment of the target muscle groups [13].The combination of deep soft tissue release through IASTM therapy and the safe and efficient low-load BFR training appears to have a beneficial impact on the recovery of symptoms in PFPS subjects.

## Research object and method

### Study object

This study has received ethical approval from the university for human subjects. Students with long-term participation in different sports programs, with knee pain or discomfort, and who meet inclusion and exclusion criteria can be selected as subjects. The focus is on requiring subjects to undergo four tests conducted by a sports rehabilitation therapist, namely: patellar tracking, passive patellar tracking test, patellar tilt assessment, and Waldron test. Completion of the tests and meeting positive criteria will result in a diagnosis of patellofemoral pain or dysfunction and qualify individuals for inclusion in the trial. This experiment has been registered on the ISRCTN platform (www.isrctn.com; 07/02/2023; ISRCTN88098928) for transparency and accountability. The specific inclusion and exclusion criteria are shown in Table 1.

**Table 1:** Exclusion and inclusion criteria

Inclusion criteria	Exclusion criteria
Age 18–35 years	Exogenous knee injuries bruises, blows, cuts, burns, contusions
Can cooperate with rehabilitation physiotherapy and training and follow-up, and will not withdraw from the study without reason	External or subcutaneous bleeding from ruptured skin of the knee joint
Patellofemoral joint pain for more than 6 weeks	Born with abnormal bone structure
Positive patellar sliding/passive sliding trajectory	Presence of knee inflammation, patellar dislocation or subluxation, ligament damage
Anterior knee pain during exercise such as walking, running and jumping, walking up and down stairs	History of static knee arthroscopy within one year
Positive assessment of lateral patellar tilt/Waldron test is positive	Injury or discomfort to other body parts
Pain in the knee joint when the lower limb is under load	Loading exercises for lower limb related movements during the experiment

### Research methodology

The control group received IASTM treatment exclusively, while the experimental group received both IASTM treatment and Blood Flow Restriction (BFR) pressure training.

#### Control group

The treatment requires the subjects to assume different positions, including: supine position with the thigh flexed, abducted, and externally rotated to release the medial head of the quadriceps femoris muscle; supine position with the knee flexed and the lower leg placed outside the bed to release the rectus femoris, vastus intermedius muscles; supine position with the knee flexed to a neutral position to release the soft tissues around the patella; lateral position with the knee flexed to release the lateral head of the quadriceps femoris muscle, tensor fasciae latae muscle; prone position to release the hamstring muscle group (with emphasis on the biceps femoris muscle in the posterior lateral aspect of the thigh). Please refer to Table 2 for specific steps.

**Table 2:** Steps in IASTM treatment

Tools	Methods	Strength	Time	Purpose
Type C—Scanning knife	Uniformly apply fascial lubricant in the treatment area, and use a C-shaped probe knife to apply pressure and slide along the direction of muscle fibers, targeting the muscle group at a 45-degree angle from either bottom to top or top to bottom	Low	1 min	The gradual adaptation of subjects to tool therapy is accompanied by the identification of areas of fascial densification or granular regions [14–16]
Type A—Shark knife	The high resistance areas of fascial densification or trigger points [17] are subjected to slow and repeated pressure sliding	Low-Medium	3-5 min	The loosening of soft tissues in stiff areas is performed in two states, namely the lower limb resting state and the maximum extension state, in order to restore the elasticity of soft tissues and alleviate or eliminate pain points
Type B—Bat-knife	Applying pressure to the treatment area at an angle of approximately 45 degrees, massage should be performed in two directions: from top to bottom and from bottom to top. In areas of fascial densification or trigger points, small areas of repeated pressure and sliding should be applied	Medium–High	3 min	A high degree of concentration and greater pressure is used to achieve deeper muscle release
Type M—Large M blade	Subjects were instructed to perform uninterrupted knee flexion–extension or leg abduction–adduction movements, while receiving passive myofascial blade pressure and sliding with coordinated breathing	Medium–High	5-8 min	Dynamic release of deep muscle groups to increase intermuscular glide and restore range of motion in joints
Type S—Hook knife	Apply pressure and sliding perpendicular to the direction of muscle fibers on local stiff areas and trigger points	Low-Medium	1-3 min	Deeper relaxation of agonising pain points

#### Test group

The experimental group received IASTM treatment combined with BFR, using pressurized equipment for training. The selected exercises included seated leg curls, standing leg curls, and weighted hip flexion squats. According to the latest literature review on the selection of exercise variables for BFR, the optimal exercise variable data includes: load 20%-50% 1RM, repetition count divided into 15–30 times/group, total exercise count of 50–80 times (e.g., 30–15-15–15 times), 3–5 sets, within-group rest time of 30–60 s (pressurized), and between-group rest time of 5 min (non-pressurized) [18]. Based on this, the exercise plan for this experiment was set accordingly. The number of exercise sets increased with the number of treatment weeks (3–6 sets). The rest time between sets was 30-60 s, and each set consisted of 15–30 repetitions [19]. Pressurized training was conducted twice a week [20]. BFR was performed half an hour after the subjects completed IASTM treatment, with the pressurized band placed at the proximal 1/3 of the thigh. Before training, the pressure was adjusted to 20 mmHg for warm-up exercises. According to the different lower limb dimensions and self-perception of the subjects, the training pressure value was adjusted to 20-50 mmHg 1RM [21], with no pressure applied during the training process. A professional coach supervised and corrected the execution of the exercises and controlled the pressure value throughout the entire process. See Table 3

**Table 3:** Specified actions of test group

Exercise	Procedure	Frequency	Intensity	Pressurization value	Progression
Seated leg extensions	During the treatment, the subject will be positioned at the side of the bed and will alternate between sitting and standing. The compression band will be placed on the upper third of the thigh, and the subject will perform knee flexion and extension exercises. To engage the muscles fully, the subject will exhale for three seconds during a centripetal contraction and inhale for three seconds during a centrifugal contraction. At the peak of the muscle contraction, the subject will hold the position for one second	2 times a week for 4 weeks	15–25 times in group 1 A total of 4–6 groups	20-50 mmHg	In the first week, there were four groups with 15 participants in each group. In the second week, there were five groups with 20 sessions per group. The third week consisted of Group 6 with 25 sessions per group. Additionally, Group 4 had six participants and underwent 25 sessions per group
Standing Leg Curl	In this exercise, the subject should stand with their thighs still and complete a retroflexion of the knee. They should exhale for 3 s during knee flexion and inhale for 3 s during knee extension. Additionally, they should hold the position for 1 s at the peak of muscle contraction	2 times a week for 4 weeks	15–25 times in group 1 A total of 4–6 groups	20-50 mmHg	4 groups in the first week, 15 in each group; 5 groups in the second week, 20 sessions per group; Group 6 in the third week, 25 sessions per group; Group 4, *n* = 6, 25 sessions per group
Weighted Hip Flexion Squat	In this subject paper, it is stated that the proper stance for a squat involves standing with your feet slightly wider than shoulder width apart, with your knees facing your toes. To perform the exercise, you should complete a hip flexion squat, inhaling for three seconds as you lower your body. Then, exhale as you extend your knees and stand up for three seconds	2 times a week for 4 weeks	15–20 times in group 1 A total of 4 groups	20-50 mmHg	4 groups in the first week, 15 in each group; 4 groups in the second week, 20 sessions per group; Group 4 in the third week, 25 sessions per group; In the fourth week in group 4, 20

### Observation indicators

#### Lysholm knee score

The Lysholm score [22] was utilized to evaluate the functional status of patients’ knee joints both pre- and post-treatment. The score encompasses eight different aspects, including pain, instability, occlusion, swelling, limp, stair climbing ability, kneeling posture, and brace usage. The total score possible is 100.The instability and pain criteria were each worth 25 points, with a survey score below 65 being considered poor. A score between 65 and 83 was qualified, while a score between 84 and 94 was considered good, and a score between 95 and 100 was excellent. The higher the score, the better the lower function, allowing for the treatment effect to be judged based on the score.

#### VAS score

The level of patellofemoral pain was assessed prior to and following treatment using the Visual Analogue Scale (VAS) [23]. This scale ranges from 0 to 10, with increasing levels of pain intensity. A score of 0 indicates no pain, while a score of 10 represents intolerable pain.

#### Maximal isometric muscle strength test of lower limb extensors

Enhancement of lower limb muscle strength and neuromuscular control can improve the functional performance of PFPS patients and reduce the wear of the patellofemoral joint [24]. Therefore, this study used the lower limb extensor maximal isometric strength testing system (model: dr.wolff sports & prevention) to conduct tests before and after treatment. This testing system can provide strong support for rehabilitation therapists in the diagnosis, evaluation, and formulation of rehabilitation programs for patients’ lower limb functional training. During the testing process, the subject was seated with their waist and hips close to the equipment, and both lower limbs were at a fixed knee joint angle of 90° for the knee extension force test. In a single strength test, the force was stopped when the value reached its peak, and a total of three tests were completed. The average of the three peak force values was calculated.

#### Modified Thomas test

**Fig. 1:**
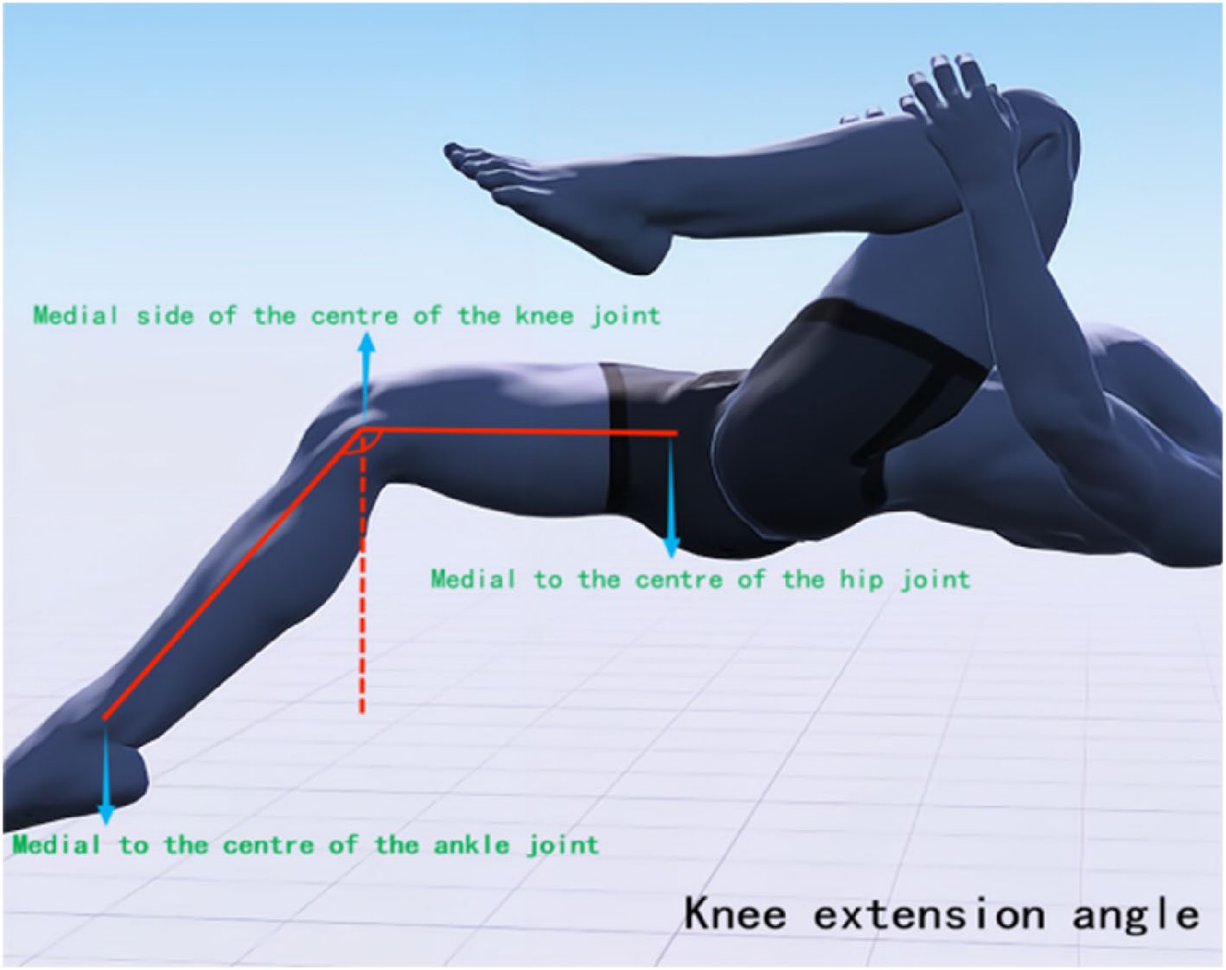
Measurement of knee extension angle

**Fig. 2:**
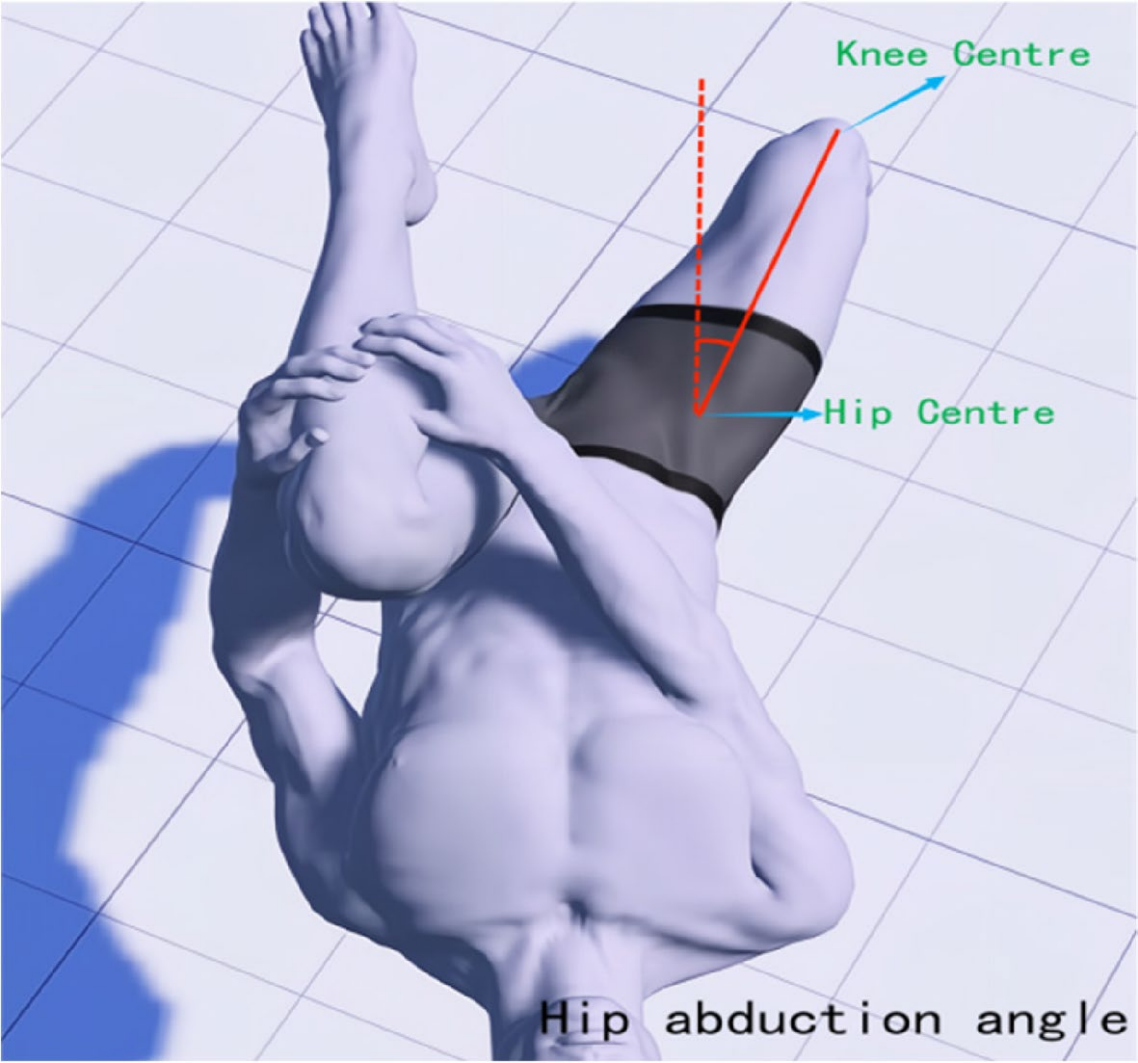
Measurement of hip abduction angle

**Fig. 3:**
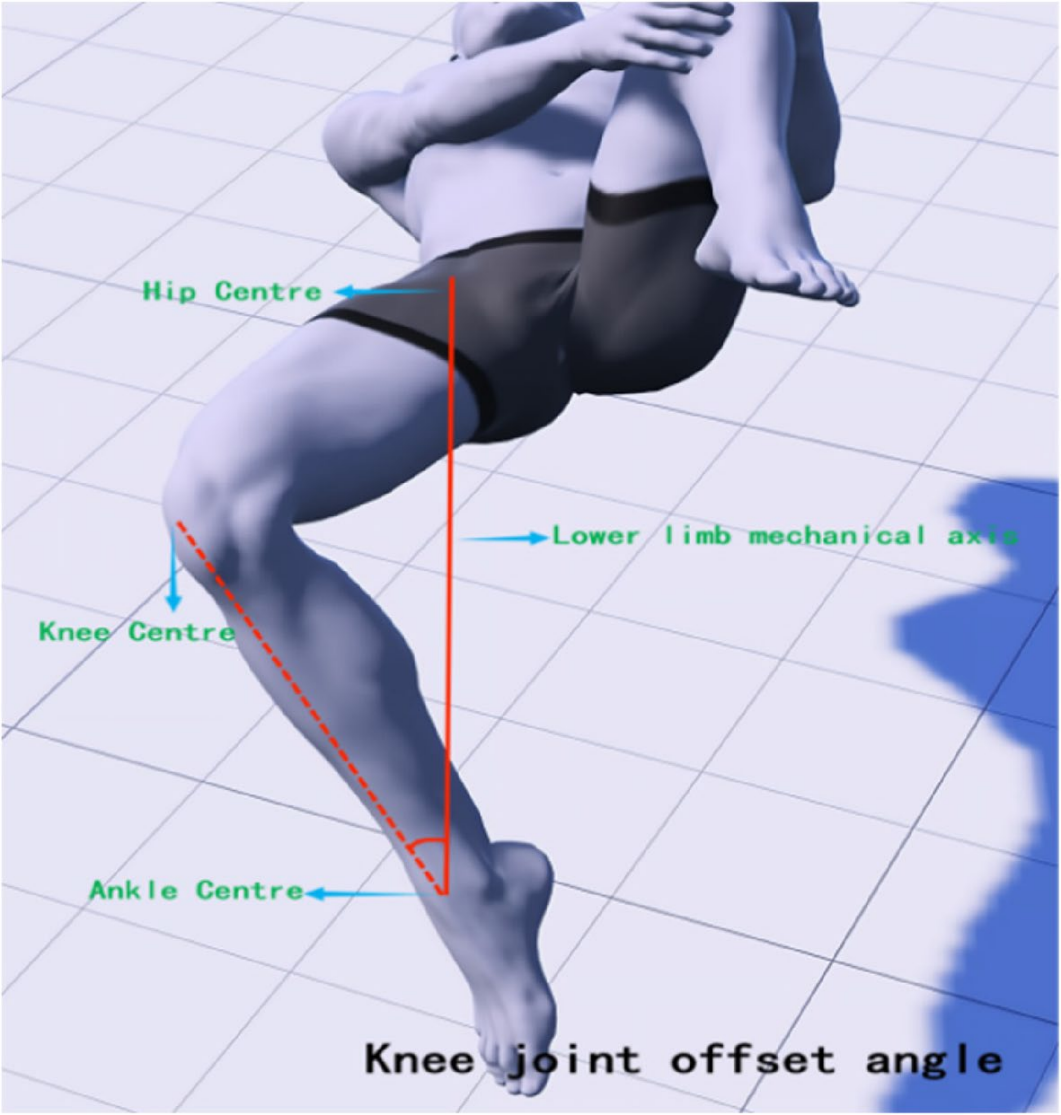
Knee Offset Angle Measurement

The Modified Thomas Test (MTT) is a method for assessing the flexibility of the iliopsoas, rectus femoris, and tensor fasciae latae muscles, and has high reliability for testing the tension of the lower limb muscles [25]. In this study, a high-precision joint mobility ruler was utilized to observe alterations in lower limb joint angles prior to treatment, immediately following the initial treatment, and after four weeks of treatment. During the test, the subject is positioned in a semi-seated position at the edge of the bed, with both hands holding the healthy side of the tibia near the knee and lying down directly. The examiner observes whether the femur is parallel to the bed surface; if there is an angle between the femur and the bed surface, it is judged that the hip flexor (iliopsoas) is shortened and tense. At this time, the state of the affected leg is observed; if the hip is excessively abducted, it is judged that the tensor fasciae latae is tense; if it is excessively adducted, it is judged that the pectineus muscle is tense; if the leg shows calf adduction and femur external rotation (similar to kicking a shuttlecock), it is judged that the hip internal rotator muscles (sartorius, semitendinosus, and semimembranosus) are tense; if the angle between the knee joint and the calf is too large, it is judged that the rectus femoris is tense. During the test, interference from iliopsoas tension is excluded, and the mechanical axis position and the hip-knee-ankle (HKA) joint center points are determined [26]. The axis is defined as the line connecting the hip joint center (aligned with the femoral major trochanter and femoral head center, located deep in the inguinal region) and the ankle joint center (midpoint of the tibia width). The test mainly focuses on recording the muscle groups affecting the knee joint status. After IASTM treatment, the MTT is performed again, and a high-precision goniometer is used to measure the knee extension angle (measured from the medial side of the knee joint, see Fig. 1), hip abduction angle (measured from the hip center, see Fig. 2), and knee joint displacement mechanical axis angle (measured from the ankle joint center, aligning with the hip joint center as the axis, see Fig. 3).

### Statistical analysis

The data in this study were analyzed and processed using SPSS 26.0 statistical software. SPSS was used to read, test, and statistically analyze the data. The general data were analyzed using the independent samples t-test. The measurement data were found to conform to a normal distribution. Additionally, the presence of three time point measurement indicators was analyzed using repeated measures ANOVA. Paired t-tests were used before and after treatment for two time point measures. *p* < 0.05 was considered a statistically significant difference.

This study used G.power 3.1.9.7 for sample size calculations, as the experimental groups were ANOVA with repeated measures and interactions, therefore, for software calculations we chose F tests, ANOVA: Repeated measures, between factors, Type of power analysis We chose A priori: Compute required sample size—given a, power, and effect size. specific parameters included; Effect size f = 0.25, α err prob = 0.05, Power (1-B err prob) = 0.8, and Number of groups = 2, Number of measurements = 3, Corr among rep measures = 0. The final sample size result was 44/3 = 14.6≈15, while the sample size for this experiment was 26 cases, in line with the results calculated by G. power.

## Results

### Baseline characteristics of participants

**Fig. 4:**
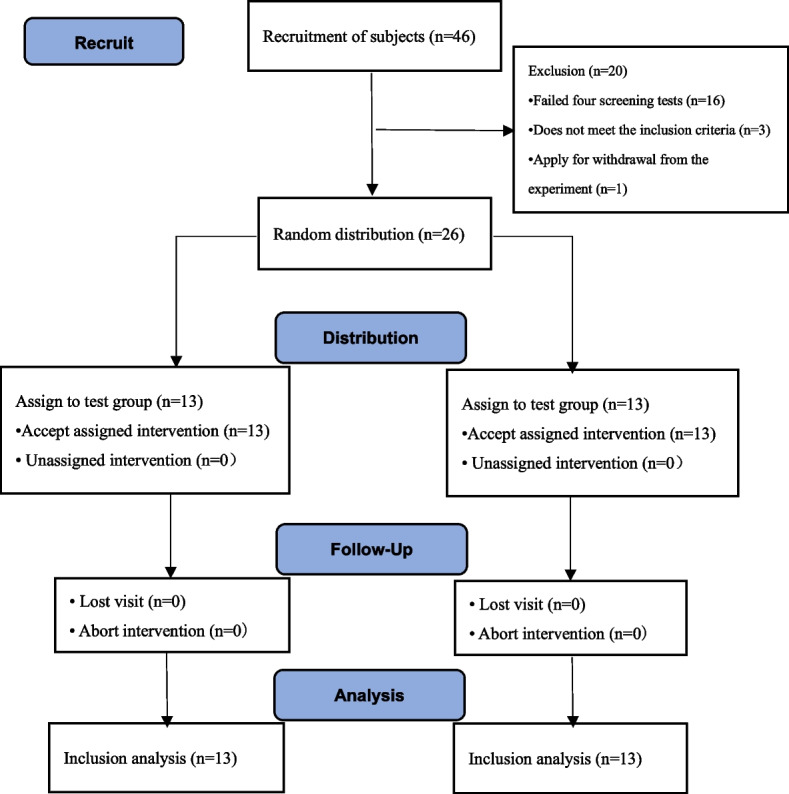
Flow chart of subject recruitment during the trial

The trial recruited a total of 46 people, and ultimately selected 26 subjects with different specializations who met the criteria for PFPS, based on exclusion and inclusion criteria. These patients were then randomly divided into control (*n* = 13) and test groups (*n* = 13) using the random number table method (see Fig. 4). There were no significant differences (*P* > 0.05) in the subjects’ gender age, height, body mass, duration of illness, (see Table 4).

**Table 4:** Baseline characteristics of participants

Projects	Control group (*n* = 13)	Test group (*n* = 13)	t	*p*
Gender (m/f, n)	5/8	11/2	-2.132	0.054
Age (years)	22 ± 2.12	20.92 ± 2.18	-1.176	0.263
Height (cm)	176.08 ± 7.24	170.54 ± 6.63	-2.274	0.042
Body mass (kg)	74.54 ± 13.87	60.71 ± 10.33	-3.619	0.004
Duration of pain (weeks)	12.92 ± 8.34	22.12 ± 19.19	1.740	0.107

General information using independent samples t-test

*n* Number

### Lysholm knee score

The ANOVA by repeated measurements of Lysholm knee at various time points was conducted to determine the impact of treatment on the scores. The results indicated that there was a significant effect of group change on Lysholm score (*F* = 4.32, *p* = 0.048), as well as a significant effect of different time point measurements on Lysholm score (*F* = 27.97, *p* < 0.001). These findings suggest that treatment had a positive effect on Lysholm scores, and that the scores varied significantly depending on the time point of measurement; However, there was no significant effect of group when comparing between them. It was noted that there was no significant change in Lysholm scores in either the test or control groups until after 4 weeks of treatment (*p* = 0.005).There was a significant difference observed between two treatments and pre-treatment after the first treatment and after 4 weeks of treatment (*p* < 0.05), which was not statistically significant. The statistical results are presented in Tables 5 and 6.

**Table 5:** Results of two-factor repeated measures ANOVA for changes in test indicators at three time points of the Lysholm rating scale

**Lysholm repeated evaluation F-test**			
	F	P	Bias η2
Group main effect	4.32	0.048	0.153
Point-in-time main effect	27.97	0.000	0.538
Group x time point	0.71	0.49	0.029

**Table 6:** Results of the comparison of the changes in test values with the mean values at three time points of the Lysholm rating scale

	**Before treatment**	**After the first treatment**	**After 4 weeks of treatment**	**Multiple are comparable**
	M ± SD	M ± SD	M ± SD	
Test group	61.62 ± 12.64	82.77 ± 18.75^*&α^	84.08 ± 9.71^*#&α^	Before treatment < after the first treatment < after 4 weeks of treatment
Control group	70.85 ± 17.20	86.00 ± 11.05^*&α^	94.54 ± 7.23^*#&α^	Before treatment < after the first treatment < after 4 weeks of treatment

Tables were analysed using repeated measures ANOVA

^*^Represents significant difference in change in Lysholm score compared to pre-treatment (*p* < 0.05)

^#^Represents significant change between test and control groups (*p* = 0.005)

^&^Represents no significant change in comparison between time points within groups (*p* > 0.05)

^α^Represents a significant change between after the first treatment and after 4 weeks of treatment (*p* < 0.05; M ± SD indicates mean ± standard deviation

### VAS scale score results

The ANOVA with repeated measurements of the VAS scale was conducted to analyze the impact of group change and time point measurements on VAS scores. The results indicated that group change did not have a significant effect on VAS scores (*F* = 0.45, *P* = 0.509). However, the study found a significant effect of different time point measurements on VAS scores (*F* = 39.20, *P* < 0.001). Additionally, the interaction of VAS scores was significantly affected by both group and time point change (*F* = 6.48, *P* = 0.004).When comparing between groups; there was a significant change in VAS scores between the test and control groups only before treatment and immediately after the first treatment (*p* < 0.05). There was a significant difference between groups when comparing immediately after the first treatment, twice after 4 weeks of therapy, and before treatment (*p* < 0.05). In contrast, there was no statistically significant difference in VAS scores between the two time points immediately after the first treatment and 4 weeks later within the test group (*p* > 0.05), and no statistically significant difference in VAS scores between the pre-treatment and immediately after the first treatment within the control group (*p* > 0.05). Tables 7 and 8 present the statistical findings.

**Table 7:** Two-factor repeated measures ANOVA results for changes in test metrics at three time points on the VAS rating scale

**VAS repeated evaluation F-test**			
	F	P	Bias η2
Group main effect	0.45	0.509	0.018
Point-in-time main effect	39.20	0.000	0.620
Group x time point	6.48	0.004	0.265

**Table 8:** Results of the comparison between the changes in test values and the mean values at three time points of the VAS rating scale

	Before treatment	After the first treatment	After 4 weeks of treatment	Multiple are comparable
	M ± SD	M ± SD	M ± SD	
Test group	23.23 ± 13.91^#^	4.00 ± 4.30^*#&α^	2.77 ± 3.22^*&α^	Before treatment > after the first treatment > after 4 weeks of treatment
Control group	14.54 ± 5.98^#&^	8.69 ± 6.37^*#&α^	2.92 ± 5.75^*α^	Before treatment > after the first treatment > after 4 weeks of treatment

Tables were analysed using repeated measures ANOVA

^*^Represents significant difference in change in VAS scores compared to pre-treatment (*p* < 0.05)

^#^Represents significant change between test and control groups (*p* < 0.05)

^&^Represents no significant change in comparison between time points within groups (*p* > 0.05)

^α^Represents a significant change between after the first treatment and after 4 weeks of treatment (*p* < 0.05); M ± SD indicates mean ± standard deviation

### Comparison of pre- and post-treatment data for maximum strength of lower limb extensors

Maximum lower extremity extensor strength was only tested before treatment versus after 4 weeks of treatment. Within-group comparison; lower extremity extensor strength increased before and after treatment in both the control and test groups, with a significant difference (*p* < 0.001). Between-group comparison; the tested strength values did not change significantly (*p* > 0.05) was not statistically significant. See Table 9.

**Table 9:** Comparison of lower limb strength data between the two groups before and after treatment

Group	*n*	Before treatment	After 4 weeks of treatment	t-value	*p*-value
Control group	13	100.77 ± 53.63	118.06 ± 52.04	-5.888	< 0.001
Test group	13	61.50 ± 37.90	79.62 ± 38.00	-6.345	< 0.001
t-value		-2.045	-2.131		
*p*-value		0.063	0.054		

Using paired t-tests

*n* Number

### Modified Thomas experiment

The results of ANOVA by repeated measurements of the MTT test at different time points before treatment, after the first treatment, and at 4 weeks of treatment showed that all three groups of angle measurements had a non-significant effect on angle change, (*F* = 0.04, *F* = 2.37, *F* = 1.39), (*P* = 0.85, *P* = 0.14, *P* = 0.25); there was a significant effect of different time point measurements on angle change, (*F* = 189.95, *F* = 119.11, *F* = 41.18), (*P* < 0.001); there was a non-significant interaction between group and time point on angle change for knee extension angle and knee offset angle, (*F* = 1.83, *F* = 1.92), (*P* = 0.17, *P* = 0.16); there was a significant interaction between group and time point on angle change for hip joint angle (*F* = 4.59, *P* = 0.03).

When comparing between groups; there was no significant change in the values of the three angle measurements between the test group and the control group before treatment and immediately after the first treatment (*p* > 0.05). In the intra-group comparison; there was a significant change in the three angle measurements between immediately after the first treatment and twice after 4 weeks of treatment, and before treatment (*p* < 0.05). In contrast, there was no significant change (*p* > 0.05) in angle measurements between the immediate after first treatment and two time points after 4 weeks of treatment for knee extension angle within the test group, and no significant change (*p* > 0.05) in angle measurements between the immediate after first treatment and two time points after 4 weeks of treatment for knee excursion angle within the control group. The statistical results are shown in Tables 10 and 11.

**Table 10:** Two-factor repeated-measures ANOVA results for joint angle change in MTT test

	Knee extension angle			Hip abduction angle			Knee joint offset angle		
	F	*P*	Bias η2	F	*P*	Bias η2	F	*P*	Bias η2
Group main effect	0.04	0.85	0.002	2.37	0.14	0.090	1.39	0.25	0.055
Point-in-time main effect	189.95	0.000	0.888	119.11	0.000	0.832	41.18	< 0.001	0.632
Group x time point	1.83	0.17	0.071	4.59	0.03	0.160	1.92	0.16	0.074

**Table 11:** MTT test joint angle value changes compared with the mean value results

Group	Projects	Before treatment	After the first treatment	After the final treatment	Multiple are comparable
		M ± SD	M ± SD	M ± SD	
Test group (*n* = 13)	Knee extension angle	123.36 ± 7.62	103.88 ± 7.35^*&^	102.26 ± 7.23^*&^	Before treatment > after the first treatment > after 4 weeks of treatment
	Hip abduction angle	29.67 ± 10.57	13.67 ± 4.92^*a^	7.13 ± 4.24^*a^	Before treatment > after the first treatment > after 4 weeks of treatment
	Knee joint offset angle	16.92 ± 10.51	7.78 ± 3.60^*a^	5.38 ± 3.13^*a^	Before treatment > after the first treatment > after 4 weeks of treatment
Control group (*n* = 13)	Knee Extension	125.69 ± 4.89	105.15 ± 4.23^*a^	99.85 ± 6.26^*a^	Before treatment > after the first treatment > after 4 weeks of treatment
	Hip abduction angle	40.21 ± 15.06	13.53 ± 6.25^*a^	8.47 ± 3.01^*a^	Before treatment > after the first treatment > after 4 weeks of treatment
	Knee joint offset angle	12.66 ± 4.72	6.12 ± 2.76^*&^	5.43 ± 2.89^*&^	Before treatment > after the first treatment > after 4 weeks of treatment

^*^Represents significant difference in angular change compared to pre-treatment in within-group comparisons (*p* < 0.05)

^&^Represents no significant change in within-group comparisons at different time points (*p* > 0.05); M ± SD indicates mean ± standard deviation

^a^Represents significant difference in angular change after first treatment and 4 weeks of treatment in within-group comparisons (*p* < 0.05)

### Long-term follow-up results

Subjects were followed up at three time points: one month, three months, and six months after the end of the trial. The study aimed to determine at what point pain would develop and how often it occurred (See Tables 12 and 13). The results showed that most people did not experience significant knee pain at the six-month mark. During the first three months, only one male subject in the test and control groups experienced occasional pain during running or weight-bearing exercise. In the control group, two female subjects experienced occasional pain during running and weight-bearing exercise at month three, respectively. At 6 months, one male and one female subject in the test group had occasional pain during weight-bearing exercise, and one male subject had frequent pain during stair climbing. In the control group, one male and one female subject had occasional pain during stair climbing, and three female subjects had occasional pain during weight-bearing exercise. In summary, only a few subjects experienced occasional knee discomfort or pain during a particular exercise session in the first six months, and most of the pain was only experienced during weight-bearing exercise and not during daily lying, sitting, standing, or walking exercises. These findings suggest that the first and middle months of the treatment intervention in both groups were effective in treating the symptoms of PFPS patients.

**Table 12:** Moments of pain follow-up results

Moments of Pain	TG/1 M	CG/1 M	TG/3 M	CG/3 M	TG/6 M	CG/6 M
Laying flat						
Walking						
Sitting and standing						
Running				m = 1, f = 1		
Load-bearing	m = 1	m = 1	m = 1	f = 1	m = 1, f = 1	f = 3
Walking up and down stairs					m = 1	m = 1,f1

*TG* Test group, *CG* Control group, *m* Male, *f F*emale, *M* Month

**Table 13:** Frequency of pain follow-up results

Frequency	TG/1 M	CG/1 M	TG/3 M	CG/3 M	TG/6 M	CG/6 M
Occasional	m = 1	m = 1	m = 1	m = 1f = 2	m = 1,f = 1	m = 1,f = 4
Frequent					m = 1	
Pain-free	m = 10, f = 2	m = 4,f = 8	m = 10, f = 2	m = 4,f = 6	m = 9, f = 1	m = 4,f = 4

*TG* Test group, *CG* Control group, *m* Male, *f* Female, *M* Month

## Discussion

The application of IASTM therapy and BFR training in the field of sports rehabilitation or musculoskeletal pain rehabilitation is not uncommon, and these two treatment methods have their own advantages. IASTM therapy can loosen deeper muscles and has a better immediate effect [27]. BFR training can use pressure devices to achieve high-load training under low-load conditions [28], and both treatment methods have been proven to improve patients’ strength and function [5, 29]. Therefore, this experiment combines the two interventions, aiming to achieve safe and efficient improvement of pain, function and strength in PFPS patients through a combination of exercise and physical interventions. After experimental intervention, the results showed that IASTM therapy combined with BFR can significantly reduce PFPS patient pain, improve knee soft tissue flexibility, increase lower limb strength, and increase joint range of motion. Pure IASTM therapy has significant effects on improving knee pain and muscle flexibility, and can make the knee joint exert greater strength in a painless state. However, in terms of overall treatment effect, the combined treatment is significantly better than pure IASTM therapy.

As the most complex joint in the body, the knee joint has a high usage rate in coordinating and stabilizing lower limb movements, which inevitably leads to injury and pain, resulting in a high incidence of knee-related disorders year-round. Most patients with knee disorders share common characteristics such as poor movement patterns, lifestyle habits and abnormal body posture. Among them, PFPS [1] has a complex and unclear pathogenesis that correlates with lower extremity functional deficits, injuries during sports, and long-term poor quality of life, and there is early evidence that abnormal pressure on the medial and lateral sides of the joint and weakness of the lower extremity joint stabilizing muscles, especially the quadriceps, hip abductors and external rotators, are associated with knee pain [30]. It is also widely accepted that knee pain may be associated with its own morphology or damaged tissues, including abnormal tension of the periprosthetic muscles and the iliotibial bundle, damaged ligaments (anterior and posterior cruciate ligaments, medial and lateral collateral ligaments), abnormal Q-angle, meniscal tears, abnormal tibial position, knee hyperextension, and limited hip motion [31–35]. However, there is no direct study to reveal the underlying cause of PFPS production. Therefore, in this study, we used IASTM treatment combined with BFR training as a therapeutic intervention for patients with PFPS by conservative treatment means to loosen the soft tissues around the knee joint and strengthen the strength of the balanced periarticular muscle groups, so as to reduce or eliminate pain, strengthen the lower limb strength, restore knee function, and improve the range of motion of the joint.

BFR training does not exert excessive joint stress on the patient. At the same time, the mechanism of action of the compression device allows for blood filling of the periarticular muscles, adequate protection of the joint, and increases the sense of muscle strength and recruitment, which has the characteristics of high safety and high training efficiency. According to Erickson [36] et al., BFR compression training helps to improve and enhance the neuromuscular recruitment of the Quadriceps Femoris Muscle (QFM) in patients with Anterior Cruciate Ligament Reconstruction (ACL-R). (QFM) neuromuscular recruitment capacity. This appears to have the same effect as IASTM treatment, and is supported by Bobes [37] et al., who showed a significant increase in QFM strength and cross-sectional area after having patients with ACL-R and knee osteoarthritis trained with BFR low load. Also Van [29] et al., by Mata analysis included eight studies implementing lower load strength training combined with BFR versus traditional QFM strength training on symptoms and function in patients with knee disorders and found that BFR increased knee extensor strength and QQFM thickness. However, traditional single quadriceps strength training has the potential to exacerbate knee symptoms [37, 38]. Therefore, in this study, BFR compression training was performed to strengthen knee extension strength in addition to knee flexor muscle groups and external rotation ability to improve knee stabilization strength in multiple dimensions.

IASTM therapy is effective in improving the patient’s joint range of motion pain and strength [5]. It is commonly used as a non-invasive treatment modality for soft tissue (skeletal muscles, ligaments, fascia) injuries and post-operative rehabilitation [6, 7]. At the same time, the pushing of the release tool on the soft tissue surface seems to activate or inhibit the fascia mesoreceptors [39], which has been shown to be effective for delayed activation of inactivated muscle groups around the knee joint. It has been shown [27] that the IASTM intervention can raise the skin temperature of the hamstrings and maintain it for more than one hour. This external pressure stimulation increases the skin blood flow and accelerates the local metabolism, providing the fascial space for muscle congestion and tension expansion, creating the conditions for muscle strength training. Therefore, the combined follow-up BFR compression training has a multiplier effect.

## Conclusion

Through the results of this study, it was found that IASTM had a significant effect immediately after the treatment, with immediate effect in improving soft tissue flexibility and, regulating the abnormal tension in the soft tissues of the stabilized joint, reducing the unbalanced stress on the knee joint, reducing the wear and tear within the joint, and creating a good mechanical environment for it, thus reducing or eliminating musculoskeletal pain. Then, the patient uses BFR training in the pain-free state of the lower limb joints, after the equipment pressure makes the lower limb as a whole, especially the knee joint, blood filling, strength increase, muscle proprioception enhancement, and training the peripheral muscle groups of the knee joint in the correct movement trajectory, which can greatly improve the efficiency and efficiency of the training and make the stability of the knee joint in three-dimensional space enhanced. In summary, IASTM treatment combined with BFR compression training improves the symptoms of patellofemoral joint pain, limited range of motion, and muscle weakness in order to improve knee mobility, strengthen the muscle strength of the flexor and extensor groups, and restore the knee joint and other functions. Ultimately, the patient’s motor ability is enhanced, and the strength and function of the lower extremity is improved to achieve the purpose of treating PFPS.

This study has certain limitations. The current sample size is relatively small, which may lead to the influence of random errors on the results, thereby reducing the reliability of the study. The study population is more biased towards the athletic population, so the research results lack some universality. In terms of intervention time, the 4-week treatment period is obviously short. Will longer-term treatment intervention have better therapeutic effects or pose certain risks? These all need to be improved in future research. In addition, the BFR training program design in the experiment is relatively single, and the training movements mainly strengthen the anterior and posterior muscles of the thigh. However, some studies have shown that hip joint restriction [40] and peripheral pain will affect knee joint movement. For many patients who cannot move autonomously, when the activity is restricted, the exercise pressure will often be distributed to other joints, and the additional stress on the joints will have a risk of injury. There is also evidence [41] that patients with knee joint pain symptoms often have hip joint function limitations, and the degree of hip joint restriction is positively correlated with the degree of knee joint lesion pain. Similarly, Earl-Boehm [42] et al. conducted non-weight-bearing training on hip strength and abdominal endurance in PFPS patients, and compared it with quadriceps strengthening training. The results showed that if PFPS patients have a high degree of pain and want to maintain a high level of function, they can improve knee joint stability and balance function by training hip joint strength and core muscle strength, which can avoid the direct involvement of painful joints and improve lower limb stability. Therefore, it can be seen that strengthening hip joint strength and improving hip joint restriction have a positive effect on the treatment of PFPS symptoms.

Based on the research foundation of this study, it is recommended that future research endeavors should aim to expand the participant pool by recruiting individuals from diverse backgrounds, ages, genders, races, and geographic locations. It is crucial to study different populations, such as elderly individuals and patients, to assess the applicability of the combined therapy’s effects in various populations, ensuring that the research findings are more representative. Additionally, collecting and analyzing more data can help understand the differences between different populations and ensure that the research conclusions are universal. To improve the design of exercise programs and movement selection, exercises involving hip joint strength training can be incorporated, and physical therapy can be utilized to alleviate hip joint restrictions. Future research can use more objective indicators, such as blood biochemical indicators, imaging indicators, hip joint strength, and functional indicators, and extend the treatment intervention period to more comprehensively evaluate the effectiveness of the combined therapy.

## Data Availability

The data underlying this paper, which includes the privacy of the individuals involved, cannot be made public for the following reasons. These data will be shared with the respective authors upon reasonable request. If you need the data, you can contact me via my email. 784389072@qq.com.
